# Carotid intima-media thickness in the Brazilian Longitudinal Study of Adult Health (ELSA-Brasil): a narrative review

**DOI:** 10.1590/1516-3180.2017.0272141017

**Published:** 2018-03-05

**Authors:** Eduardo Henrique Sena Santos, Pedro José dos Santos, Itamar de Souza Santos

**Affiliations:** I MD. Physician, Imaging Service, and Researcher, Center for Clinical and Epidemiological Research, Hospital Universitário (HU), Universidade de São Paulo (USP), São Paulo (SP), Brazil.; II MD, PhD. Researcher, Center for Clinical and Epidemiological Research, Hospital Universitário (HU), Universidade de São Paulo (USP), and Associate Professor, Department of Internal Medicine, Faculdade de Medicina da Universidade de São Paulo (FMUSP), São Paulo (SP), Brazil.

**Keywords:** Carotid intima-media thickness, Atherosclerosis, Cardiovascular system, Risk factors, Cross-sectional studies

## Abstract

**BACKGROUND:**

Carotid intima-media thickness (CIMT), as measured by ultrasound, has been used in large studies as a non-invasive marker for subclinical atherosclerosis. The Brazilian Longitudinal Study of Adult Health (ELSA-Brasil) is a cohort of 15,105 civil servants in six Brazilian cities that included CIMT evaluation in its baseline assessment. The aim of the present narrative review was to provide an overview of ELSA-Brasil CIMT articles published up to July 31, 2017.

**DESIGN AND SETTING:**

Narrative review of ELSA-Brasil CIMT studies using baseline assessment data.

**METHODS:**

We searched PubMed for the terms “ELSA-Brasil” and “intima-media”. This search yielded 21 published articles using CIMT data from the ELSA-Brasil baseline assessment, which were included in this review. We also present information about intima-media thickness assessment from ongoing onsite reevaluations of the study participants.

**RESULTS:**

Most published studies focused on the association with traditional and novel cardiovascular risk factors. Studies also presented information about the ELSA-Brasil CIMT protocol at baseline and CIMT value distribution in this large sample.

**CONCLUSIONS:**

Analyses on the ELSA-Brasil data led to important insights on CIMT interpretation and physiology. Besides the highlighted contributions which have already been made in this field, new data gathered during the ongoing third onsite assessment will enable investigation of substantially new research questions.

## INTRODUCTION

Intima-media thickness (IMT), as measured by ultrasound, has been used in both observational and intervention studies as a non-invasive marker for subclinical atherosclerosis.^1^ It has been shown to be associated with cardiovascular risk factors,^2^ and to be a predictor of cardiovascular events.^3^ Carotid IMT (CIMT) progression over time is used as a surrogate outcome for atherosclerotic disease in clinical trials.^4^

Ultrasound examination allows adequate characterization of carotid walls, is easily executed, is less costly than other imaging techniques, and is widely available. CIMT can be measured by means of B-mode ultrasound, and is defined as the distance between two interfaces: (a) the vascular and intima lumina; and (b) the middle and adventitial layers.^1^ Despite many efforts that have been made towards standardizing CIMT definitions, there is no complete consensus in the literature about the best anatomical location, the number of measurements, or which summary of measurements (mean or maximum CIMT) should be used.

The Brazilian Longitudinal Study of Adult Health (ELSA-Brasil)^5^^,^^6^ is a cohort study on 15,105 civil servants aged 35 to 74 years, in six Brazilian cities (São Paulo, Belo Horizonte, Rio de Janeiro, Porto Alegre, Salvador and Vitória). CIMT data were collected at baseline between August 2008 and December 2010. ELSA-Brasil has one of the largest CIMT datasets among observational studies. As a reference, the ELSA-Brasil CIMT sample size is comparable to large, iconic cohorts that included CIMT evaluation, such as the Atherosclerotic Risk in Communities (ARIC)^7^ study and the Multi-Ethnic Study of Atherosclerosis (MESA),^8^ and is larger than other important CIMT samples such as the Northern Manhattan (NOMAS)^9^ and Rotterdam^10^ studies.

The aim of the present narrative review was to provide an overview of articles on CIMT in ELSA-Brasil cohort articles published prior to July 31, 2017, and to summarize the contributions that these studies have made to current knowledge.

## METHODS

We searched PubMed for the terms “ELSA-Brasil” and “intima-media” to select the articles included in this review. ELSA-Brasil has a steering committee and a publications and presentations committee.^11^ Both are composed of researchers from each ELSA-Brasil investigation site and also keep track of these published papers, which were added to this review.

We classified these articles according to their main objectives, into three subsections: “CIMT protocol and value distributions at ELSA-Brasil baseline”, “CIMT and traditional cardiovascular risk factors” and “CIMT and novel cardiovascular risk factors or other conditions”. We also present information about IMT assessment from ongoing onsite reevaluations of study participants (ELSA-Brasil Visit 3) and some of the study perspectives for the near future.

## RESULTS

We found 21 published articles using CIMT data from the baseline assessment. **Table 1** shows the main aims, findings and conclusions of articles focusing on the association with traditional risk factors and **Table 2** shows the main aims, findings and conclusions of articles focusing on the association with novel cardiovascular risk factors or other conditions.

**Table 1:** ELSA-Brasil studies evaluating the association between carotid intima-media thickness and traditional cardiovascular risk factors

Main aims and associations	Main findings	Main conclusions
Age, sex and race^12^	Median regression slopes: +0.0069 to +0.0079 (per one-year increase in age)	CIMT increased with age in all sex and race groups.
	AdjB for male sex: +0.036 (P < 0.001)	CIMT was higher in men than in women.
	AdjB for mixed race (compared with blacks): -0.030 (P < 0.001)	Black race was associated with higher CIMT values than those of whites. Mixed-race individuals had intermediate values, but these were closer to those found in whites.
	AdjB for white race (compared with blacks): -0.041 (P < 0.001)	
AHA CVH score^13^	AdjB for one-point increase in the CVH score: -0.011 (95% CI: -0.012 to -0.010)	CIMT values were significantly and inversely associated with the CVH score.
Blood pressure variability^14^	Standardized path coefficient from BP variability to residual CIMT (after regression to assess main confounders): +0.046 (P < 0.001)	After minimizing the influence of the main confounders, there was a small but significant association between systolic blood pressure variability and CIMT values.
Glucose levels 10 to 12 years before baseline^15^	AdjB for glucose levels 110-125 mg/dl: +0.028 (95% CI: +0.003 to +0.053)	Participants with glucose levels between 110 and 125 mg/dl in 1998 had higher CIMT values at the ELSA-Brasil baseline than did those with glucose levels < 100 mg/dl.
	AdjB for incident diabetes: +0.034 (95% CI: +0.015 to +0.053)	Participants with incident diabetes during follow-up had higher CIMT values at the ELSA-Brasil baseline than did those without incident diabetes.
HALP^16^	AdjB for HALP: -0.020 (95% CI: -0.042 to +0.003)	CIMT values were not associated with HALP after adjustment for confounders.
Neck circumference^17^	AdjOR for the association between one SD increase in NC and CIMT ≥ 75^th^ percentile	NC was associated with CIMT but not CAC, thus suggesting that fatty tissues possibly had a local effect.
	Men: 1.66 (95% CI: 1.28-2.14)	
	Women: 1.52 (95% CI: 1.16-1.99)	
Resistant hypertension^18^	Prevalence ratio for resistant hypertension associated with higher CIMT values: 3.15 (95% CI: 2.09-4.74)	Individuals with resistant hypertension had higher CIMT values than did those with non-resistant hypertension.
Smoking^19^	AdjB for current smokers: +0.03 (95% CI: +0.02 to +0.04)	Current and former smokers had higher CIMT values.
	AdjB for former smokers: +0.01 (95% CI: +0.01 to +0.02)	
	Interaction between age and current smoking: +0.013 (P < 0.001)	The association between current smoking and CIMT was stronger among older individuals, males, non-whites and individuals with lower educational levels.
	Interaction between female sex and current smoking: -0.30 (P < 0.001)	
	Interaction between race and current smoking: +0.062 (P = 0.03)	
	Interaction between educational level and current smoking: -0.087 (P = 0.004)	
Variance explained by traditional risk factors^20^	R^2^ for main models: 0.141 to 0.373	Less than 40% of CIMT variance was explained by traditional risk factors.
	AdjB for one SD increase in blood pressure (all individuals)	Pulse pressure, LDL/HDL ratio and neck circumference were the most consistent contributors to the main models, while the association with measurements of glucose metabolism was weaker.
	Men: 0.017 (P < 0.001); women: 0.023 (P < 0.001)	
	AdjB for one SD increase in LDL/HDL ratio (all individuals)	
	Men: 0.009 (P < 0.001); women: 0.009 (P < 0.001)	
	AdjB for one SD increase in NC (all individuals)	
	Men: 0.026 (P < 0.001); women: 0.019 (P < 0.001)	
	AdjB for one SD increase in glycohemoglobin (all individuals)	
	Men: 0.005 (P < 0.05); women: 0.006 (P < 0.01)	

95% CI = 95% confidence interval; AdjB = adjusted beta-coefficient; AdjOR = adjusted odds ratio; AHA = American Heart Association; CIMT = carotid intima-media thickness; CVH = cardiovascular health; HALP = hyperalphalipoproteinemia. HDL = high-density lipoprotein; LDL = low-density lipoprotein; NC = neck circumference; SD = standard deviation.

**Table 2:** ELSA-Brasil studies evaluating the association between carotid intima-media thickness and novel cardiovascular risk factors, and other association studies

Main aims and associations	Main findings	Main conclusions
Adiponectin^21^	AdjOR for the association with CIMT ≥ 75^th^ percentile for a given age, sex and race:	Low adiponectin levels were associated with CIMT values in this subgroup, after adjustment for multiple confounders (including body mass index).
	Log-transformed adiponectin: 0.78 (95% CI: 0.63-0.97)	
Ankle-brachial index^22^	Positive likelihood ratios for high CIMT, using:	Positive likelihood ratios for high CIMT (≥ 75^th^ percentile) were higher when the highest (compared with mean or lowest) ankle systolic BP was used for ankle-brachial index calculation.
	Highest ankle systolic BP: 2.79 (95% CI: 1.50-5.18)	
	Mean ankle systolic BP: 2.68 (95% CI: 1.74-4.13)	
	Lowest ankle systolic BP: 1.83 (95% CI: 1.42-2.36)	
	Negative likelihood ratios for high CIMT, using:	Negative likelihood ratios for high CIMT (≥ 75^th^ percentile) were similar for all ankle-brachial index calculation strategies.
	Highest ankle systolic BP: 0.99 (95% CI: 0.99-1.00)	
	Mean ankle systolic BP: 0.99 (95% CI: 0.98-1.00)	
	Lowest ankle systolic BP: 0.98 (95% CI: 0.97-0.99)	
Cognitive performance^23^	AdjB for delayed word recall test score: -0.433 (95% CI: -0.724 to -0.142)	CIMT values were inversely associated with performance in memory tests.
Cognitive performance^24^	Significant path coefficients for the indirect path between HRV and the TMT-B via HOMA-IR and CIMT:	Both insulin resistance and CIMT values mediated the association between heart rate variability and performance in the TMT-B.
	Path coefficient for HRV to HOMA-IR: −0.0942 (P < 0.001)	
	Path coefficient for HOMA-IR to CIMT: +0.0340 (P < 0.0001)	
	Path coefficient for CIMT to TMT-B: +0.1052 (P < 0.0001)	
Endothelial function^25^	AdjB for the reactive hyperemia index: +0.060 (P = 0.023)	Endothelial function (according to peripheral arterial tonometry) was inconsistently associated with CIMT. Endothelial dysfunction and CIMT may represent distinct phenomena or different stages of the atherosclerotic process.
	AdjB for the mean basal pulse amplitude: +0.010 (P = 0.221)	
HIV infection^26^	AdjB for HIV group: 0.004 (95% CI: -0.006 to +0.014)	Comparing ELSA-Brasil data with information from individuals with HIV infection (mostly undergoing combined antiretroviral therapy), CIMT values did not differ between groups after adjustment for sociodemographic variables and cardiovascular risk factors.
Insulin resistance^27^	AdjOR for the association with CIMT ≥ 75^th^ percentile for a given age, sex and race:	There was a direct association between insulin resistance and CIMT values, while glucose levels or glycated hemoglobin were not associated with CIMT in the main models. This raised the hypothesis that a direct effect from insulin on atherosclerosis or insulin-promoted medial hypertrophy may be involved.
	One SD increase in HOMA-IR: 1.10 (95% CI: 1.04-1.17)	
Mental symptoms^28^	OR for the association with CIMT ≥ 75^th^ percentile for a given age, sex and race:	Intensity and frequency of mental symptoms, along with generalized anxiety disorder and common mental disorder, were associated with higher CIMT values in full models.
	For one SD increase in CIS-R scores: 1.12 (95% CI: 1.06-1.19)	
	For common mental disorder disease: 1.22 (95% CI: 1.07-1.38)	
	For generalized anxiety disorder: 1.19 (95% CI: 1.01-1.41)	
Migraine^29^	AdjB for migraine with aura: -0.01 (95% CI: -0.03 to +0.01)	Migraine, regardless of the presence of aura symptoms, was not associated with CIMT values after adjustment for confounders.
	AdjB for migraine without aura: -0.01 (95% CI: -0.02 to +0.01)	
Socioeconomic mobility^30^	AdjB for downward intergenerational mobility (3 or more levels in a 7-level ladder): +0.013 (P = 0.04)	Downward intergenerational mobility was associated with higher CIMT values, and the more intense the downward mobility was, the more intense this association also was. For intragenerational mobility, individuals with stable low social status had higher CIMT values than did those with stable high social status.
	AdjB for stable low social status: +0.012 (P = 0.03)	
Socioeconomic status^31^	AdjB for low life course socioeconomic position:	CIMT values were positively associated with more prolonged exposure to low socioeconomic status (cumulative lifetime socioeconomic position).
	Men: +0.049 (95% CI: +0.020 to +0.079)	
	Women: +0.031 (95% CI: +0.001 to +0.063)	
Subclinical hypothyroidism^32^	AdjB for subclinical hypothyroidism: +0.010 (95% CI: +0.001 to +0.019)	Subclinical hypothyroidism was associated with CIMT values after adjustment for major confounders.
	AdjOR for the association with CIMT ≥ 75^th^ percentile for a given age, sex and race:	
	Subclinical hypothyroidism: 1.30 (95% CI: 1.06-1.59)	

95% CI = 95% confidence interval; AdjB = adjusted beta-coefficient; AdjOR = adjusted odds ratio; BP = blood pressure; CIMT = carotid intima-media thickness. HOMA-IR = homeostasis model assessment - insulin resistance; OR = odds ratio; SD = standard deviation; TMT-B: trail-making test B.

## DISCUSSION

### CIMT protocol and value distributions at ELSA-Brasil baseline

Acquisition of CIMT images in the ELSA-Brasil study complied with the recommendations from the American and Brazilian Societies of Echocardiography. All patients were examined by technicians and/or physicians who had previously been trained and certified for this protocol. The images were obtained using Toshiba Aplio XG ultrasound devices with a linear 7.5 MHz transducer. All images obtained were sent to an examination reading center in São Paulo. To measure CIMT, each common carotid artery (CCA) was identified along its longitudinal axis, using standard brightness and contrast. IMT was calculated automatically using the Medical Imaging Applications software (MIA, Coralville, Iowa, USA), with analysis on three electrocardiographically gated cardiac cycles. Only the proximal far wall of the CCA (1 cm proximally to the carotid bifurcation, with 1 cm length) was measured, and mean and maximum values for each CCA were obtained.^33^

The current Brazilian recommendations for ultrasound evaluation on carotid atherosclerotic disease use the ELSA-Brasil CIMT values as the standard for the Brazilian adult population.^34^ These standards are important, because a CIMT value ≥ the 75^th^ percentile for an individual’s age, sex and race/ethnicity, and which has been determined from the distribution in his/her reference population, is usually considered indicative of higher subclinical atherosclerosis burden.^35^

Comparing IMT values across cohorts is difficult. Differences in study populations, sample selection, year of recruitment, CIMT protocols and statistical choices influence these comparisons. Considering the context of these limitations, Santos et al.^12^ showed that for the same sex, age and race, the CIMT values in ELSA-Brasil were on the whole slightly lower than those in the ARIC study, but were similar to the findings from the German Gutenberg Heart Study.

### CIMT and traditional cardiovascular risk factors

The abovementioned article by Santos et al.^12^ also assessed some important associations for the Brazilian population. A considerable proportion (43%) of Brazilians reported themselves as “mixed race” in the 2010 national census. This high rate of racial blending, which is also reflected in the ELSA-Brasil sample, cannot be found in other large CIMT samples. These authors concluded that black individuals had higher CIMT values than did white or mixed-race individuals. CIMT values for individuals who self-reported their race as mixed were closer to those found for whites than to those for blacks, after adjustment for age and sex. These findings may reflect constitutional aspects of this measurement, but may also be a marker for racial health inequalities.

New insights about the interpretation of CIMT values came from a 2015 ELSA-Brasil article by Santos et al.^20^ The authors found that traditional cardiovascular risk factors explained less than 40% of CIMT variance in the ELSA-Brasil sample, even after adopting analytical strategies to optimize this proportion. This finding is in line with results from other samples, and suggests that there is room to analyze novel risk factors for atherosclerosis in determining CIMT values. It is possible that other factors that are not directly linked to atherosclerosis progression may influence CIMT values. For example, some of these factors may be associated with medial hypertrophy, a condition that may increase CIMT values as well. Identification of these additional factors that are unrelated to atherosclerosis is important for understanding CIMT and its association with cardiovascular risk.

Additional analyses investigating the association between CIMT values and cardiovascular health (CVH) scores at the ELSA-Brasil baseline have corroborated these interpretations. The CVH score was proposed by the American Heart Association in 2010^36^ as a tool for measuring CVH in populations. It has well-defined criteria for diet, physical activity, body mass index, smoking, blood pressure, fasting plasma glucose and total cholesterol score. Significant inverse associations were found between CVH scores and CIMT values at the ELSA-Brasil baseline, although some participants with optimum CVH scores had unexpectedly high CIMT values.^13^

Another finding from the ELSA-Brasil data was the close relationship between CIMT values and neck circumference (NC).^17^ NC was associated with CIMT values but not with coronary artery calcium, an alternative marker for subclinical atherosclerosis. Moreover, after adjustment for CCA vessel diameters, this association remained significant, thus suggesting that this does not merely result from larger CCA arteries in individuals with greater NC. One putative explanation for this finding is that there may be a paracrine effect from fatty tissue in the neck, which would influence carotid atherosclerosis but would not have an impact on arteries in other regions.

An association between CIMT values and blood pressure (BP) has been described in the ELSA-Brasil cohort^20^ and in other samples.^37^ Another two ELSA-Brasil articles have explored additional aspects of this association. Lotufo et al.^18^ analyzed data from ELSA-Brasil participants who were using antihypertensive medications. These authors found that those with resistant hypertension (defined as non-controlled BP despite using at least three different classes of antihypertensive medication or controlled BP through using at least four different classes of antihypertensive medication) had higher CIMT values than did those with non-resistant hypertension, thus suggesting that a dose-response relationship existed between BP and CIMT. However, this association is not limited to individuals using antihypertensives. Recently, Ribeiro et al.^14^ analyzed the association between CIMT values and the patterns in the ten BP measurements that were made during the six-hour baseline visit among ELSA-Brasil participants who were not using antihypertensive drugs. They found that both the central trend (as a weighted mean) of the systolic BP measurements and the systolic BP variability (standard deviation of BP measurements) were associated with CIMT values, after minimizing the influence of other traditional cardiovascular risk factors. This finding suggests that high short-term BP variability has a role in CIMT values and, potentially, in atherosclerosis.

Sitnik et al.^15^ analyzed data from a subset of ELSA-Brasil participants at the São Paulo investigation site (N = 1536) who had participated in a workplace screening assessment in 1998 and did not have diabetes at that time. One aim of that study was to evaluate whether glucose levels in 1998 or incident diabetes between assessments would predict CIMT levels at the ELSA-Brasil baseline, 10 to 12 years later. They found that participants with incident diabetes between assessments had higher CIMT values at the ELSA-Brasil baseline. Individuals with glucose levels between 110 mg/dl and 125 mg/dl in 1998 also had higher CIMT values at the ELSA-Brasil baseline than did those with glucose levels below 100 mg/dl, although this association lost significance when individuals who developed diabetes were excluded (P = 0.093).

Elevated high-density lipoprotein cholesterol (HDL-c) levels are typically considered to provide a protective cardiovascular profile. However, there is evidence linking hyperalphalipoproteinemia (HALP), a condition related to very high HDL-c levels, to higher cardiovascular risk,^38^ probably due to abnormal HDL-c activity. Using the very large CIMT dataset from the ELSA-Brasil baseline, Laurinavicius et al.^16^ found no evidence of higher CIMT values in individuals with HDL-c levels above 90 mg/dl, compared with those with normal HDL levels, in adjusted models.

Recently, Kianoush et al.^19^ further explored the association between smoking status and a set of inflammatory and atherosclerotic markers, including CIMT. Besides noting the fact that current and former smokers have higher CIMT values, these authors also suggested that this association is stronger among individuals over 50 years of age, males, non-whites and individuals with lower educational levels. They also found an association between secondhand smoking and CIMT values in models adjusted for age, sex, race and other cardiovascular risk factors. However, this association only maintained borderline significance after adjustment for participants’ smoking status and pack-years (P = 0.06).

### CIMT and novel cardiovascular risk factors or other conditions

One of the greatest strengths of the ELSA-Brasil cohort is that it enables studies on the effects of social variables on clinical (biological) characteristics. Two ELSA-Brasil studies have focused on the relationship between socioeconomic position and trajectory and CIMT. Camelo et al.^31^ evaluated the relationship between CIMT and socioeconomic position over the course of life, as measured by the participants’ jobs in their first and current occupations. They found that the association between CIMT and socioeconomic position over the course of life followed a cumulative model, in which CIMT levels were positively associated with more time spent performing jobs of low socioeconomic level. High work stress and passive work were not associated with CIMT, regardless of socioeconomic level. A different analytical strategy was adopted by Guimarães et al.,^30^ who also analyzed intergenerational social mobility. In this study, in comparing the change in occupational social class of the head of the household between the participant’s occupation at the time of starting to work and his or her current occupation, the participants with downward mobility had higher CIMT values. Moreover, the association was more intense for individuals with greater downward mobility.

Endothelial dysfunction is an important phase of the atherosclerotic process^39^ that can be measured using peripheral arterial tonometry (PAT). In a subset of participants at the Minas Gerais investigation site, Lemos et al.^25^ evaluated the association between PAT measurements and CIMT values. These authors did not find any consistent associations, thus suggesting that information from PAT and CIMT may be complementary and may represent different phenomena or stages of the atherosclerotic process.

Novel information about CIMT pathophysiology and interpretation resulted from two recent ELSA-Brasil studies. Santos et al.^27^ showed that insulin resistance was more strongly associated with CIMT values than with glucose levels, thus suggesting that a hormonal effect on CIMT may exist. This may arise through a direct effect from insulin on the pathogenesis of atherosclerosis. On the other hand, hyperinsulinemia (consequential to insulin-resistant states) may also cause medial hypertrophy, which is included in the intima-media complex. This second hypothesis is further supported by findings from de Almeida-Pititto et al.,^21^ who studied the association between CIMT values and serum adiponectin in a subsample of 687 ELSA-Brasil participants in São Paulo aged 35 to 54 years, without diabetes or cardiovascular disease at baseline. Adiponectin also inhibits vascular smooth muscle cell proliferation, and these authors found higher CIMT values in individuals with lower adiponectin levels. In addition, both higher insulin resistance and lower adiponectin levels have been correlated with visceral (including perivascular) adiposity, and we speculate that these may be potential mediators because of their association with higher cardiovascular risk.

Mental disorders and clinical cardiovascular disease are two frequently coexistent conditions, with putative bidirectional causality between them.^40^ The ELSA-Brasil baseline forms a good scenario for studying the association between subclinical atherosclerosis and mental disorders, since this baseline assessment included a validated Portuguese-language version of the Clinical Interview Schedule - Revised (CIS-R) questionnaire. CIS-R addresses non-psychotic mental symptoms and enables diagnoses in accordance with the tenth version of the International Classification of Diseases (ICD-10). Santos et al.^28^ found that higher CIS-R scores (reflecting more frequent and/or more intense non-psychotic mental symptoms) and a diagnosis of generalized anxiety disorder were associated with higher CIMT values in adjusted models.

Vascular disease is a well-known cause of dementia, but there is conflicting evidence for the association between subclinical carotid atherosclerosis and cognitive decline. Suemoto et al.^23^ studied the association between CIMT and cognitive performance among individuals without a medical history of stroke at the ELSA-Brasil baseline. These authors found that there was an association between poorer performance in a memory test (the delayed word recall test) and higher CIMT values, after adjustment for sociodemographic variables, cardiovascular risk factors, diagnosed depression and thyroid function. In addition, Kemp et al.^24^ found that CIMT mediates the association between heart rate variability (an index of cardiac autonomic function) and performance in the trail-making test B (an executive cognitive function test), thus further supporting the link between cardiovascular diseases and cognitive performance. One strength of these analyses was that they included a large number of younger adults, a population that had been poorly studied previously.

One continuing controversy is the association between migraine and cardiovascular diseases. Goulart et al.^29^ studied the association between a diagnosis of migraine (defined using a validated questionnaire based on the International Headache Society criteria) and subclinical atherosclerosis at the ELSA-Brasil baseline among participants at the São Paulo investigation site. There were no significant associations between migraine (with or without aura) and CIMT values. These results were consistent when subclinical atherosclerosis was defined according to coronary artery calcium (CAC) scores.

Hypothyroidism is associated with a poorer cardiovascular profile,^41^ but the effects of subclinical thyroid dysfunction on atherosclerosis is a matter for debate. Peixoto de Miranda et al.^32^ studied the association between CIMT values and subclinical hypothyroidism (high thyroid-stimulating hormone with normal free thyroxine levels) at the ELSA-Brasil baseline. In adjusted models, individuals with subclinical hypothyroidism at the baseline had significantly higher CIMT values than did those with normal thyroid function.

Miname et al.^22^ compared different protocols for calculating the ankle-brachial index (ABI, a measurement of peripheral artery disease) at the ELSA-Brasil baseline. ABI is based on the ratio of systolic BP measured at the ankle divided by systolic BP measured in the arm. These authors found that when the highest ankle systolic BP was used for ABI calculation, the association with CIMT values was stronger than if they used the mean or lowest ankle systolic BP.

The ELSA-Brasil CIMT data were also used to evaluate the atherosclerotic burden associated with HIV infection. Pacheco et al.^26^ compared data from HIV-positive patients who were followed in a cohort in Rio de Janeiro, HIV-negative friends of these subjects, and ELSA-Brasil participants. These authors reported that there were no significant associations between HIV infection status and CIMT values in adjusted models.

### ELSA-Brasil Visit 3 and perspectives 

The third onsite assessment (Visit 3) of the ELSA-Brasil participants began in early 2017, and is expected to end in 2018. New questionnaires, clinical and laboratory examinations, and CIMT and femoral IMT are included in the Visit 3 protocol. These new data will make it possible to study baseline characteristics that have influenced CIMT progression over an eight-year period. Furthermore, concomitant evaluation of the carotid and femoral arteries may enable greater understanding of how associations with IMT values vary in different arterial beds. Along these lines, some studies^42^^,^^43^^,^^44^ have shown that cardiovascular risk factors associated with increased CIMT are associated with IMT values at other sites, such as the femoral arteries, but the magnitudes of these associations are heterogeneous. The current approach will allow deeper exploration of relevant scientific questions raised in previous ELSA-Brasil published data, such as the potential paracrine effect of NC on CIMT, for example. On the other hand, other factors that are only weakly associated with CIMT may be more important for determining femoral IMT values.

ELSA-Brasil was designed as a cohort study. One very important research question that longitudinal ELSA-Brasil data may help to answer concerns the extent to which CIMT values and CIMT progression may predict cardiovascular events. Most current data suggest that CIMT values have higher importance than does CIMT progression in cardiovascular risk prediction.^3^^,^^45^ However, conflicting data exists,^46^^,^^47^ at least partially due to different study populations and heterogeneous CIMT protocols. In addition, plaque analyses (including total plaque area information) for the common carotid, carotid bulb and internal carotid arteries at the baseline are currently underway. Cross-sectional and longitudinal analyses using these data may be available soon.

## CONCLUSIONS

ELSA-Brasil is one of the largest studies with CIMT data. Most published studies have focused on associations with traditional and novel cardiovascular risk factors. Analyses on the ELSA-Brasil data have also led to important insights regarding CIMT interpretation and physiology. In addition to the contributions highlighted here that have already made to this field, new data gathered during the ongoing third onsite assessment will enable investigation of substantially new research questions.
